# Spectroscopic Identification of Trifluorosilylphosphinidene and Isomeric Phosphasilene and Silicon Trifluorophosphine Complex

**DOI:** 10.1021/acs.inorgchem.4c00135

**Published:** 2024-04-09

**Authors:** Guohai Deng, Marc Reimann, Carsten Müller, Yan Lu, Martin Kaupp, Sebastian Riedel

**Affiliations:** † Institut für Chemie und Biochemie−Anorganische Chemie, Freie Universität Berlin, Fabeckstrasse 34/36, Berlin 14195, Germany; ‡ Institut für Chemie, Theoretische Chemie/Quantenchemie, Technische Universität Berlin, Sekr. C7, Strasse des 17. Juni 135, Berlin 10623, Germany

## Abstract

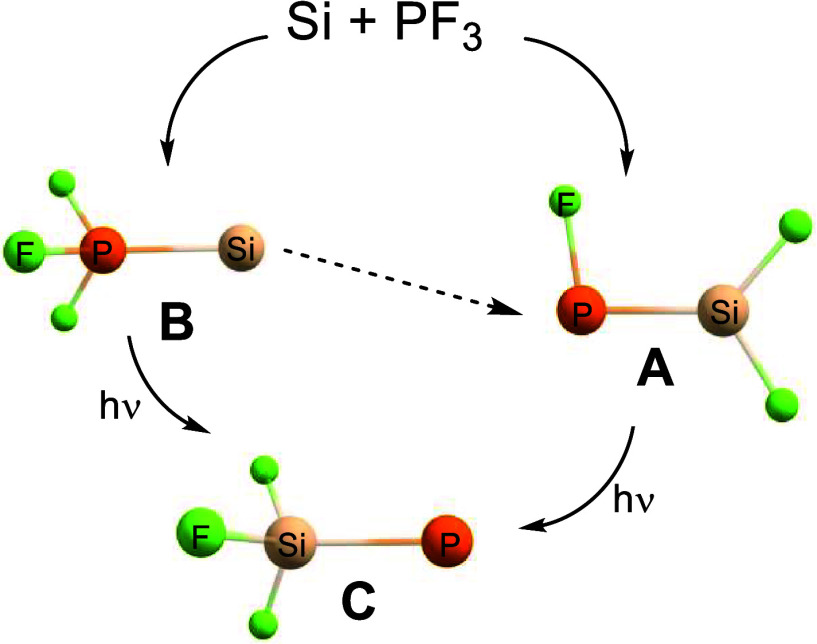

The perfluorinated silylphosphinidene, F_3_SiP, in the triplet ground state is generated by the reaction of laser-ablated silicon atoms with PF_3_ in solid neon and argon matrices. The reactions proceed with the initial formation of a silicon trifluorophosphine complex, F_3_PSi, in the triplet ground state, and a more stable inserted phosphasilene, FPSiF_2_, in the singlet ground state upon deposition. The trifluorosilylphosphinidene was formed through F-migration reactions of FPSiF_2_ and F_3_PSi following a two-state mechanism under irradiation with visible light (λ = 470 nm) and full arc light (λ > 220 nm), respectively. High-level quantum-chemical methods support the identification of F_3_PSi, FPSiF_2_, and F_3_SiP by matrix-isolation IR spectroscopy.

## Abstract 2

Three isomers with the formula F_3_PSi (**A**−**C**) were observed directly by matrix-isolation spectroscopy. The phosphasilene **A** and silicon trifluorophosphine complex **B** are formed upon co-condensation of silicon atoms and trifluorophoshine PF_3_. Subsequent irradiation leads to trifluorosilylphosphinidene **C**.

## Introduction

**Scheme 1:**
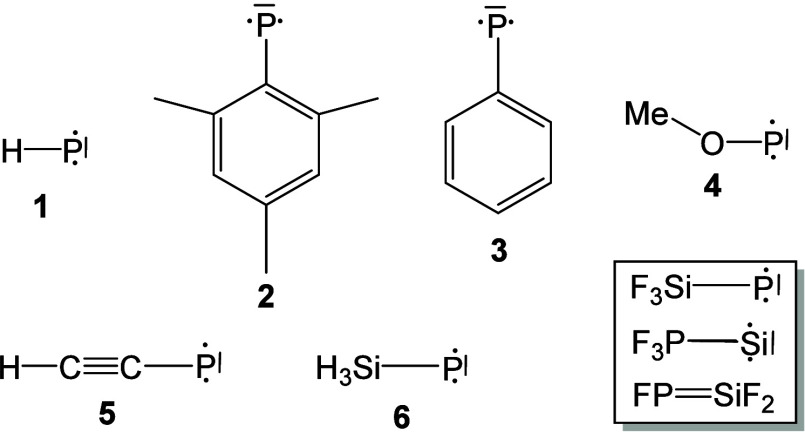
Directly Observed Uncomplexed Triplet Phosphinidenes 1–5, as well as Trifluorosilylphosphinidene F_3_Si–P and Its Two Isomers, Present Work

Phosphinidenes (R–P) are highly electron-deficient species that feature monovalent phosphorus analogues of nitrenes (R–N)^1^ and carbenes (R–C–R′).^2^ As low-valent phosphorus species, they have been widely used in synthetic chemistry as in situ phosphorus agents^3−5^ or as ligands in transition metal complexes.^6^ Due to their high reactivity and instability, fundamental knowledge about phosphinidenes is mainly achieved by trapping and complexation experiments.^7−11^ In contrast to the free nitrenes and carbenes, which have been extensively investigated,^12^ only a handful of uncomplexed phosphinidenes have been experimentally identified (Scheme 1). The parent phosphinidene (H–P) (**1**) was observed in the argon matrix following the photolysis of phosphaketene, HPCO.^13^ Mesitylphosphinidene (Mes–P) (**2**) and phenylphosphinidene (Ph–P) (**3**) were produced under matrix-isolation conditions through photoelimination of ethylene from the corresponding phosphirane and were spectroscopically characterized.^14−16^ Methoxyphosphinidene (CH_3_O–P) (**4**) was generated in a cryogenic neon matrix from the photolysis or flash-vacuum pyrolysis of methoxydiazidophosphine, and its isomeric methylphosphinidene oxide was detected.^17^ The production of ethynylphosphinidene (HCC–P) (**5**) from the dehydrogenation of phosphapropyne (CH_3_CP) via the 1-phosphapropadiene, CH_2_=C=PH, and ethynylphosphine, HCCPH_2_, intermediates through UV-light-induced rearrangement has been reported as well.^18^ The simplest silylphosphinidene (H_3_Si–P) (**6**) was formed in an argon matrix experiment via reactions of atomic silicon with phosphane.^19^ Recently, singlet (phosphino)phosphinidenes (R_2_P=P),^20^ stable at room temperature, and transient aminophosphinidenes (R_2_N=P)^21^ have been structurally characterized in the solid state and observed by mass spectrometry, respectively. In addition, the singlet diphosphinylidene H_2_P≡P was also prepared and characterized by matrix-isolation IR spectroscopy.^22^ All other directly observed free phosphinidenes have triplet electronic ground states.

It should be stressed that no experimental data have been reported on main-group fluorinated phosphinidenes so far. Trifluorosilylphosphinidene, F_3_Si–P, the perfluorinated silylphosphinidene, has been theoretically studied several times.^23,24^ It was found that phosphinidene F_3_Si–P is the most stable species of four F_3_PSi isomers in both the singlet and triplet states due to the strong Si–F bond formation. Previous reports have shown that metal phosphides, P≡MF_3_ (Cr, Mo, W, and U),^25,26^ and triplet metal phosphinidenes, F_3_M–P (Ti, Zr, Hf, and Th),^27,28^ can be formed from the reactions of laser-ablated metal atoms with trifluorophosphine. Herein, we report the production and spectroscopic characterization of triplet trifluorosilylphosphinidene via the reaction of laser-ablated silicon atoms with PF_3_ in solid neon and argon matrices. We describe that the reactions proceed with the initial formation of a silicon trifluorophosphine complex, F_3_PSi, and the more stable inserted isomer FPSiF_2_ upon deposition. The F_3_SiP molecule was synthesized through F-migration reactions of FPSiF_2_ and F_3_PSi under irradiation at visible light (λ = 470 nm) and full arc light (λ > 220 nm), respectively.

## Results and Discussion

**Figure 1:**
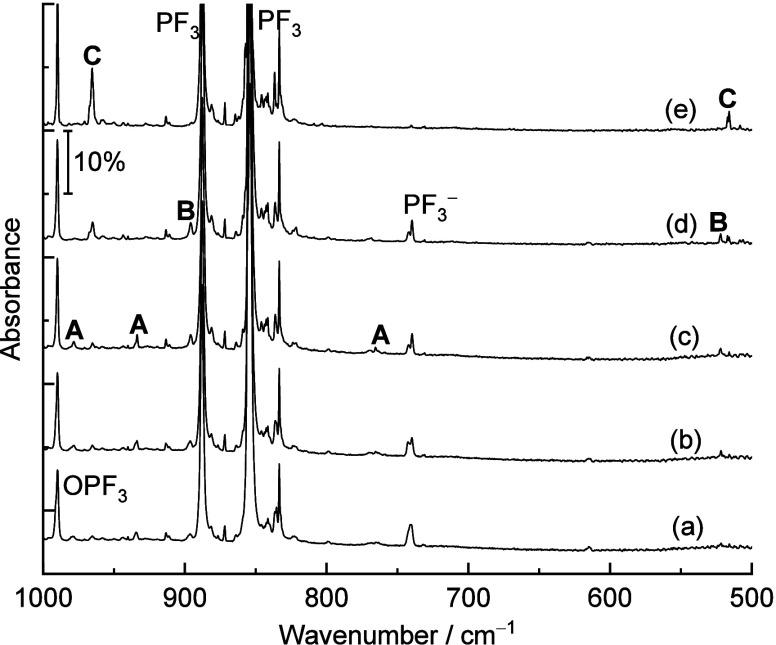
Infrared spectra in the 1000–500 cm^–1^ region from codeposition of laser-ablated Si atoms with 0.05% PF_3_ in neon. (a) After 30 min of sample deposition, (b) after annealing to 8 K, (c) after annealing to 10 K, (d) after 10 min of blue LED (λ = 470 nm) light irradiation, and (e) after 10 min of full arc (λ > 220 nm) irradiation. **A**: FPSiF_2_; **B**: F_3_PSi; **C**: F_3_SiP.

**Figure 2:**
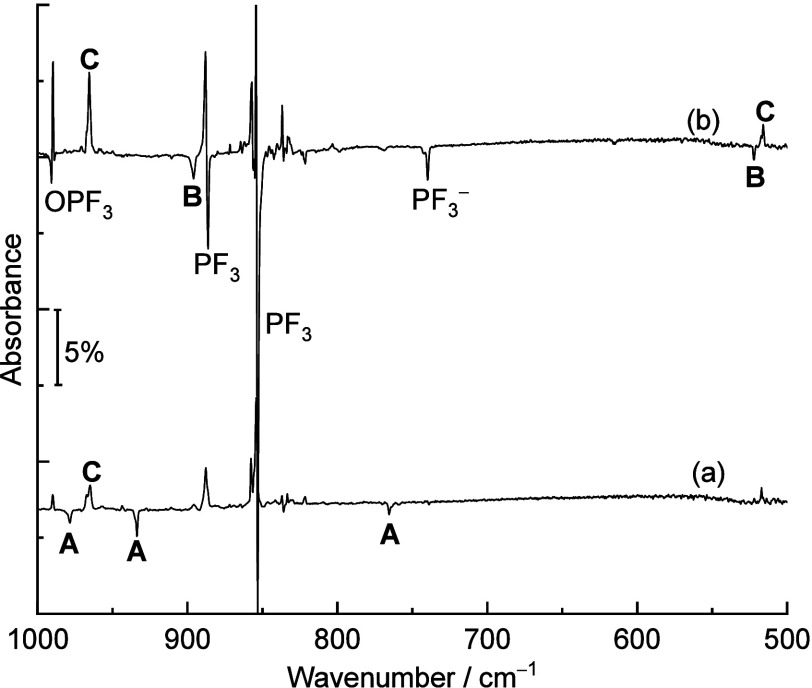
Difference infrared spectra in the 1000–500 cm^–1^ region from codeposition of Si atoms with 0.05% PF_3_ in solid neon. (a) Spectrum recorded after 10 min of blue LED (λ = 470 nm) light irradiation minus spectrum recorded after 10 K annealing. (b) Spectrum recorded after 10 min of full arc (λ > 220 nm) irradiation minus spectrum recorded after 10 min of blue LED (λ = 470 nm) light irradiation. **A**: FPSiF_2_; **B**: F_3_PSi; **C**: F_3_SiP.

The F_3_PSi, FPSiF_2_, and F_3_SiP molecules are produced via the reaction of laser-ablated silicon atoms with PF_3_ in solid neon and argon matrices. The infrared spectra in the 1000–500 cm^–1^ region obtained by using a 0.05% PF_3_/Ne sample are demonstrated in Figure 1. After 30 min sample deposition at 5 K, strong absorption bands of PF_3_^–^ (740.4 and 470.9 cm^–1^)^28^ are detected, which decrease on annealing and disappear completely upon full arc light excitation. No obvious PF_2_, SiF_2_, or SiF_3_ absorption bands were observed.^29−31^ Besides these known absorption bands, new product absorption bands were detected as well. These absorption bands can be classified into three groups according to their identical chemical behaviors (**A**, **B**, and **C** in Figures 1 and 2). The difference infrared spectra showing the photochemical transformation are given in Figure 2. Similar experiments were repeated by using a 0.2% PF_3_/Ar sample. The infrared spectra in the selected region are shown in Figure S1; the corresponding difference infrared spectra are presented in Figure S2. The band positions are summarized in Table 1.

**Table 1:** Experimentally Observed and Calculated IR Frequencies (cm^–1^) of FPSiF_2_, F_3_PSi, and F_3_SiP Molecules (Absorption Bands above 400 cm^–1^ Are Listed)

	observed		calculated	
species	Ne	Ar	CCSD(T*)-F12^a^	modes^b^
FPSiF_2_ (**A**)	978.5	974.2	977 (195.5)	asym. SiF_2_ str.
	933.7	933.1	933 (255.0)	sym. SiF_2_ str.
	765.4	759.4	765 (152.0)	P–F str.
	c	c	521 (0.4)	P–Si str.
F_3_PSi (**B**)	895.9	891.0	902 (178.3)^d^	asym. PF_3_ str.
	e	e	886 (402.7)	sym. PF_3_ str.
	522.1	520.4	521 (107.5)	P–Si str.
F_3_SiP (**C**)	965.5	959.7	965 (1560.0)^d^	asym. SiF_3_ str.
	e	e	855 (248.9)	sym. SiF_3_ str.
	516.0	513.6	514 (76.7)	Si–P str.

a Anharmonic frequencies calculated at the CCSD(T*)-F12a/aug-cc-pVTZ-F12 level; the complete sets of vibrational frequencies are provided in Supporting Information Table S1. Intensities in parentheses were obtained from HF dipole moments.

b Assignments based on calculated vibrational displacement vectors.

c Absorption band not observed due to small intensity.

d The component of the formally degenerate vibration with higher intensity is given.

e Absorption band not observed due to overlap with the PF_3_ band.

Product **A** has three absorption bands at 978.5, 933.7, and 765.4 cm^–1^. They are observed right after deposition and almost do not change when annealing to 10 K but completely disappear under blue LED (λ = 470 nm) light irradiation. Two absorption bands at 891.0 and 520.4 cm^–1^ are observed for product **B**. Both increase slightly under blue LED (λ = 470 nm) light irradiation but disappear upon full arc (λ > 220 nm) irradiation. Product **C** has two absorption bands at 965.5 and 516.0 cm^–1^. These absorption bands almost do not change upon annealing but remarkably increase under blue LED (λ = 470 nm) light and full arc (λ > 220 nm) irradiation at the expense of the absorption bands of products **A** and **B**, respectively. The product absorption bands in the argon matrix are located at 974.2, 933.1, 759.4 cm^–1^ (**A**), 891.0, 520.4 cm^–1^ (**B**), and 959.7, 513.6 cm^–1^ (**C**). All bands are red-shifted to lower wavenumbers with respect to their positions in the neon matrix due to the larger polarizability of the argon atom.

Considering the previously reported reactions of silicon atoms with PH_3_ to form the H_3_PSi, H_2_PSiH, HPSiH_2_, and H_3_SiP molecules,^19^ and of transition metal atoms with PF_3_ to give PMF_3_ complexes,^25−28^ four possible isomers F_3_PSi, F_2_PSiF, FPSiF_2_, and F_3_SiP in the electronic singlet and triplet states were studied computationally at the CCSD(T*)-F12a/aug-cc-pVTZ-F12 level.

Products **A** and **B** can be assigned to the phosphasilene isomer, FPSiF_2_, and the silicon trifluorophosphine isomer, F_3_PSi, respectively, by comparison with the computed IR spectra (Table 1 and Table S1). For product **A**, the first two absorption bands belong to antisymmetric and symmetric stretching vibration modes of the SiF_2_ moiety. The two frequencies are very close to that of F_2_Si=S (996, 969 cm^–1^, Ar matrix)^32^ but lower than that in SiF_2_ (864.6, 851.0 cm^–1^, Ne matrix).^30^ The band at 765.4 cm^–1^ can be attributed to the P–F stretching vibration, which is red-shifted compared to FP=S (791.4 cm^–1^, Ar matrix),^33^ FP=O (811.4 cm^–1^, Ar matrix),^34^ and FP=NF (826.5 cm^–1^, Ar matrix).^35^ The band positions are in excellent agreement with the computed anharmonic IR frequencies at 977, 933, and 765 cm^–1^ for the singlet FPSiF_2_ molecule. The predicted P–Si stretching vibration at 521 cm^–1^ is too weak to be observed experimentally.

For product **B**, the absorption band at 895.9 cm^–1^ belongs to the antisymmetric vibration mode of PF_3_. This position is very close to the bands of trifluorphosphine and lower than those of transition metal trifluorophosphine complexes.^36^ The P–Si stretching vibration occurs at 522.1 cm^–1^ as a weak absorption band. The band position is slightly blue-shifted compared to F_3_SiPH_2_ (514 cm^–1^, gas phase).^37^ The calculated anharmonic IR frequencies at 902 and 521 cm^–1^ of triplet silicon trifluorophores match the experimental values very well. The PF_3_ symmetric stretching vibration was predicted at 886 cm^–1^ as a strong absorption band and could not be observed due to the overlap with the respective band of PF_3_. The other computed vibrations of FPSiF_2_ and F_3_PSi are outside the present mid-IR spectral range (4000–450 cm^–1^).

Experimentally, **C** is produced under the irradiation of products FPSiF_2_ (**A**) and F_3_PSi (**B**). The reactive intermediates generally rearrange to more stable structural isomers or decompose to stable products upon photolysis. In this case, the absence of FP/SiF_2_ and P/SiF_3_ species among the photolysis products indicates that the P–Si bond in both products was not cleaved when the matrix samples were subjected to light irradiation. This is in accordance with the large P–Si bond dissociation energies of FPSiF_2_ (33.6 kcal mol^–1^, CCSD(T*)-F12a/aug-cc-pVTZ-F12) and F_3_SiP (68.3 kcal mol^–1^). Therefore, it is very likely that F-migration happens in matrix-isolated FPSiF_2_ (**A**) and F_3_PSi (**B**) under irradiation conditions. Accordingly, species **C** is safely assigned to the triplet trifluorosilylphosphinidene, F_3_SiP, the most stable isomer of F_3_PSi. Its predicted vibrational frequencies (Table 1) are in excellent agreement with the detected IR spectra. The SiF_3_ antisymmetric stretching mode appears at 965.6 cm^–1^, which is very close to those of SiF_3_ (958.6 cm^–1^, Ne-matrix)^30^ and F_3_SiPH_2_ (970 cm^–1^, gas phase).^37^ The weak band at 516.0 cm^–1^ is assigned to the Si–P stretching vibration mode, which is blue-shifted to 522.1 cm^–1^ in the F_3_PSi complex. However, the computed SiF_3_ symmetric vibration at 855 cm^–1^ is not detected due to its overlap with the PF_3_ bands in this region. The calculated vibrational frequencies of the proposed isomer F_2_PSiF (see Table S1) do not match any experimentally observed IR bands. As the agreement between theory and experiment is excellent for the other isomers, we conclude that no significant amount of F_2_PSiF is present at any given time.

**Figure 3:**
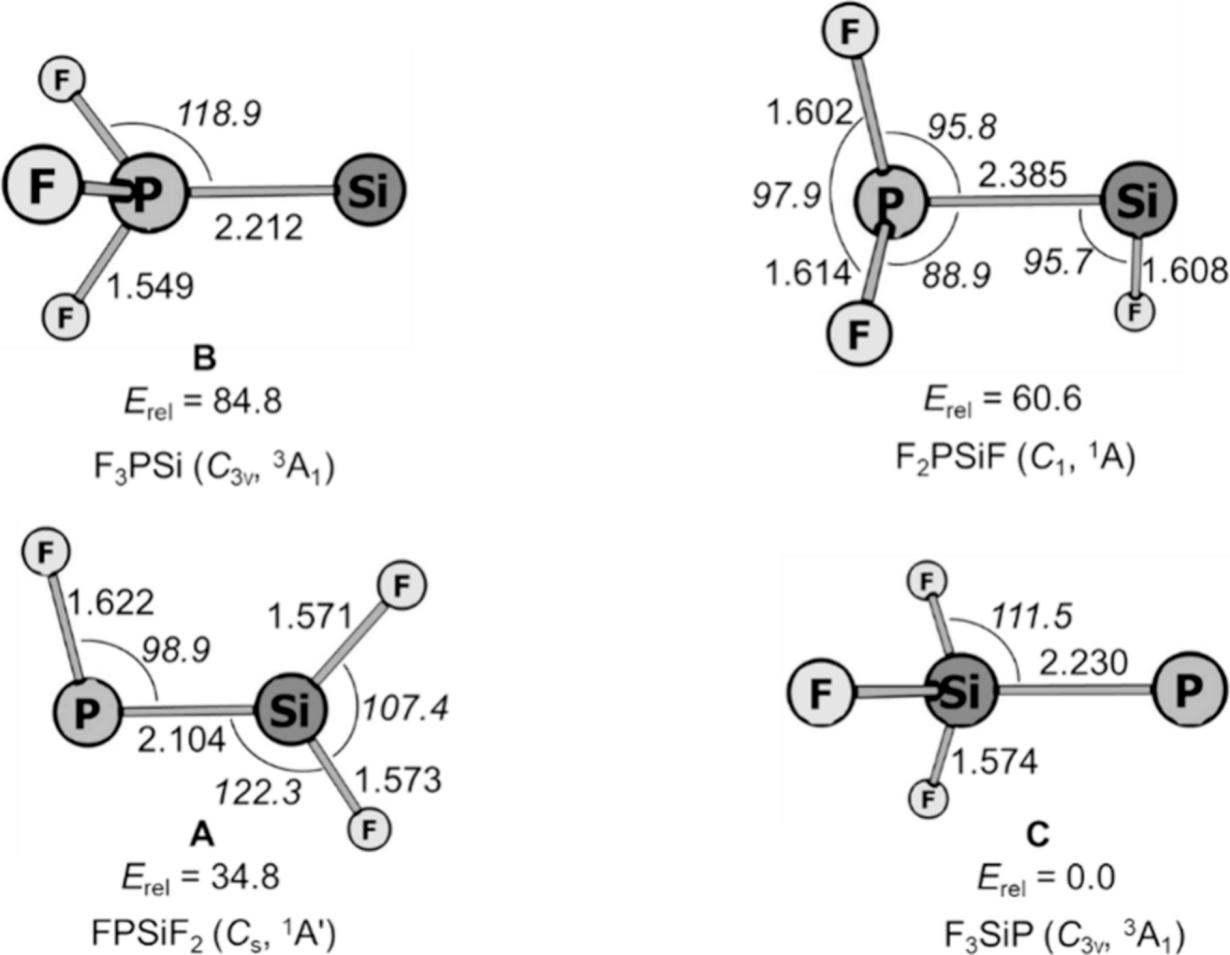
Calculated structures (bond lengths in Ångstrom and bond angles in degrees) and relative energies (kcal mol^–1^) of four F_3_PSi isomers at the CCSD(T*)-F12a/aug-cc-pVTZ-F12 level of theory.

The relative energies and structures of F_3_PSi isomers **A**, **B**, and **C** determined by ab initio calculations (CCSD(T*)-F12a/aug-cc-pVTZ-F12) are shown in Figure 3. The FPSiF_2_ (**A**) species was predicted to have a singlet ground state with a planar structure. The calculated Si–P bond length is 2.104 Å, which is close to the value (2.094 Å) determined by X-ray crystallography on phosphasilenes, but slightly longer than those of HPSi (2.045 Å)^38^ and HPSiH_2_ (2.084 Å).^19^ The analysis using the AdNDP method^39^ on FPSiF_2_ shows one Si–P σ bond and one Si–P π bond (Figure S3), suggesting the Si–P double bonding character. The calculated Wiberg bond index for the Si–P bond is 1.71, consistent with the AdNDP results. Still, the Si–P bond in FPSiF_2_ is weaker than the formal single bond in F_3_SiP (see above). NBO^40^ analysis suggests that this is due to a significant population of the Si–P π* bond due to negative hyperconjugation.

Silicon trifluorophosphine complex **B**, which has not been discussed in earlier theoretical studies, possesses, according to calculations and experiments, a triplet ground state and *C*_3v_ symmetry. The energetically higher-lying singlet silicon trifluorophosphine has *C*_1_ symmetry and lies 15.2 kcal mol^–1^ above **B**. The computed Si–P bond length (2.212 Å) is larger than in FPSiF_2_ (2.119 Å) but shorter than in the H-analogue H_3_PSi (2.356 Å).^19^

Similar to other phosphinidenes, such as HP,^13^ C_6_H_5_P,^14^ CH_3_OP,^17^ and H_3_SiP,^19^ which have been characterized by a triplet ground state, the trifluorosilylphosphinidene (**C**) is predicted to have a ^3^A_1_ ground state with *C*_3v_ symmetry. The calculated Si–P bond length is 2.230 Å, which is larger than the values of FPSiF_2_ (2.119 Å) but close to that of the F_3_PSi (2.250 Å) complex, calculated at the same level of theory. Si–P multiple bonding is absent. The calculated singlet–triplet energy gap Δ(*E*_ST_) of trifluorosilylphosphinidene is 25.0 kcal mol^–1^. As the most stable isomer of F_3_PSi, the triplet trifluorosilylphosphinidene, F_3_SiP (**C**), lies −34.8, –60.6, and −84.8 kcal mol^–1^ below FPSiF_2_ (**A**), F_2_PSiF, and F_3_PSi (**B**), respectively.

**Figure 4:**
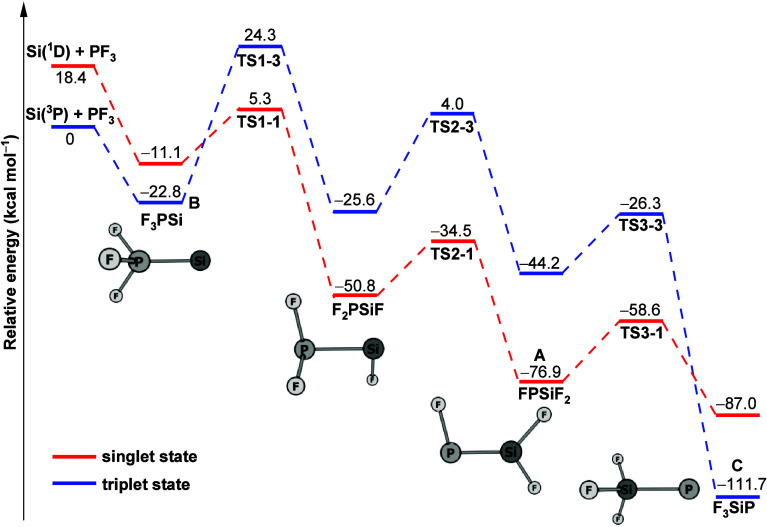
Potential energy profile along the Si + PF_3_ reaction path calculated at the CCSD(T*)-F12a/aug-cc-pVTZ-F12//B3LYP/aug-cc-pVTZ level. Energies are given in kcal mol^–1^ relative to the reactants Si(^3^P) and PF_3_.

To unravel the reaction mechanism, the potential energy profile for the reaction of silicon atoms with PF_3_ was calculated in both singlet and triplet states at the CCSD(T*)-F12a/aug-cc-pVTZ-F12//B3LYP/aug-cc-pVTZ level. The results are summarized in Figure 4. The structures of the corresponding intermediates and transition states are displayed in Figure S4. Similar to the reaction of a silicon atom with PH_3_,^19^ the first step of the formation of F_3_PSi, starting from a Si atom and trifluorophosphine, is predicted to be exothermic and barrier-free.

If the initial silicon atom has a triplet spin state, we suspect the formation of product **B**. As further barriers on the triplet surface are high, we expect no further reactions at this point. A singlet Si should lead to the formation of singlet F_3_PSi, which then reacts thermally to product **A** by fluorine transfer. The intermediately formed F_2_PSiF is not isolated as both barriers (TS1–1 and TS2–1) in the process are of similar height. Inspection of the D_1_ diagnostics^41^ for the CCSD wave functions of the singlet species shows unsuspicious values between 0.01 and 0.05 for every species but the second transition state (TS2–1). For the latter, D_1_ surpasses 0.20, indicating a stronger multireference character. This can be rationalized by the fact that FPSiF_2_ (product **A**) contains a Si–P double bond, while F_2_PSiF does not. The transition state between the two is therefore an intermediate between a Si–P single and double bond, giving rise to the importance of at least two different configurations, thereby suggesting a multireference character. As this will give rise to correlation-energy contributions of TS2–1 beyond what is captured at the CCSD(T*)-F12a level, the calculated barrier shown in Figure 4 is an upper bound. Additional calculations with a cc-pVDZ basis set suggest a further lowering of the barrier at TS2–1 compared to that at CCSD(T) by 2 kcal/mol when applying the higher CCSDT(Q) level. The effect on the other barriers is about one order of magnitude smaller. Possibly, further reductions may be expected at levels with even more static correlation. In any case, this barrier is significantly smaller than those at either TS1–1 or at TS3–1. Keeping in mind that energy dissipation in an Ar matrix is much smaller than for reactions in solution yet significant compared to a gas-phase reaction,^42^ this is suggested to explain the sole formation of **A** in the thermal reaction. That is, we have to assume that upon formation of **A**, energy dissipation is already so large that the last, higher barrier at TS3–1 cannot be overcome thermally. The computed result is consistent with our experimental observation that only very little product **A** is formed overall.

STEOM-CCSD/aug-cc-pVTZ calculations find a (formally spin-forbidden) singlet–triplet excitation for product **A** at around 465 nm. The formed triplet state can then cross TS3 on the triplet surface and relax to give the final product **C**. This explains the experimentally observed transition from **A** to **C** upon irradiation with blue light. At the same level, the first triplet–triplet excitation of **B** is found only at 390 nm with several more excitations present below 220 nm. Upon irradiation with full arc light, several excited-state pathways will therefore be accessible, allowing for the formation of thermodynamically stable product **C**.

## Conclusions

In conclusion, we report the reactions of laser-ablated silicon atoms and PF_3_ forming phosphasilene FPSiF_2_ (**A**) and silicon trifluorophosphine complex F_3_PSi (**B**) in solid neon and argon matrices. The phosphasilene molecule has an electronic singlet ground state, while the silicon trifluorophosphine complex has a triplet ground state. The FPSiF_2_ (**A**) and F_3_PSi (**B**) molecules rearrange to the more stable trifluorosilylphosphinidene F_3_SiP (**C**) molecule by one- and three-fluorine atom migration under irradiation at visible light (λ = 470 nm) and full arc light (λ > 220 nm), respectively. The latter molecule is identified to have a triplet electronic ground state.

## Experimental and Computational Details

The experimental method for matrix-isolation infrared spectroscopy has been described in more detail in our previous works.^43^ Briefly, the 1064 nm fundamental of a Nd:YAG laser (Continuum, Minilite II, 10 Hz repetition rate with 10 ns pulse width) with a pulse energy of up to 50–60 mJ cm^–2^ was used to ablate a rotating silicon target (abcr, 99.999%) to form Si atoms. The produced Si atoms were codeposited with PF_3_ (abcr, 99%) (0.05%) in excess of neon (0.2% in argon) onto a gold-plated mirror cooled to 4K using a closed-cycle helium refrigerator. After 30 min of sample deposition, infrared spectra were recorded on a Bruker Vertex 80 spectrometer at 0.5 cm^–1^ resolution in the region between 4000 and 450 cm^–1^ using a liquid nitrogen-cooled mercury cadmium telluride (MCT) detector. The matrix samples were subjected to irradiation with visible light using a LED light (Oslon 80 4+ PowerStar Circular 4 LED Arrays: λ = 470 ± 20 nm) and a medium-pressure mercury arc streetlamp (λ > 220). No uncommon hazards are noted.

Density functional theory (DFT) calculations were carried out using the Gaussian 16 program package.^44^ The hybrid functional B3LYP^45−48^ was applied in our calculations to obtain molecular structures of minima and transition states. All stationary points were characterized by the appropriate number of imaginary frequencies. All transition states were further analyzed using an intrinsic reaction coordinate calculation and were confirmed to connect the correct minima of the potential energy surface.

The Coupled Cluster calculations with Single, Double, and perturbative Triple substitutions CCSD(T) were carried out in the closed-shell (RHF-CCSD(T)) and partially spin-restricted open-shell (RHF-RCCSD(T)) formalism using default frozen core settings as implemented in the Molpro2022 software package.^49^ To approach the basis set limit, explicitly correlated calculations were performed using the F12a approximation.^50,51^ Explicit correlation effects on the perturbative triples were estimated by scaling the (*T*) contribution as
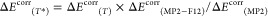


All calculations were performed using aug-cc-pVTZ-F12 basis sets^52^ as well as the respective auxiliary basis sets automatically assigned by the MOLPRO program.

Anharmonic vibrational frequencies were calculated at the VCISDTQ56 level of theory^53,54^ allowing up to five excitations within one mode. VCI calculations were performed on a polynomial fit^55^ of a multilevel surface^56,57^ using CCSD(T*)-F12a/aug-cc-pVDZ-F12 energies for the two-body and CCSD-F12a/aug-cc-pVDZ-F12 energies for the three-body terms. Intensities were computed using dipole moments at the HF level of theory.

Bonding analyses were carried out by using the adaptive natural density partitioning (AdNDP) method. The color-mapped AdNDP isosurface (0.5 au) graphs were rendered by the VMD 1.9.3 program.^58^ Additionally, natural-bond orbital (NBO) analyses were used. Optical excitation energies were obtained at the STEOM-CCSD^59,60^ level. Calculations were performed using the ORCA program,^61^ Version 5.0.2. All calculations employed the RIJCOSX approximation,^62^ the aug-cc-pVTZ basis sets,^63^ and the appropriate auxiliary basis sets.^64,65^ In every case, the first 10 excitation energies were obtained. Additional CCSDT(Q) calculations were performed using the MRCC program package and the cc-pVDZ basis set.^66^

## Supporting Information Available

Infrared spectra from co-deposition of silicon atoms with PF_3_ in argon; difference infrared spectra from co-deposition of Si atoms with PF_3_ in argon; AdNDP chemical bonding and nonbonding pattern of FPSiF_2_; calculated structural parameters of the higher-energy isomers and the transition states; calculated total energies and IR frequencies for various F_3_PSi isomers; and the Cartesian coordinates of the studied complexes (PDF)
