# Supplementary material for: Spectroscopic Identification of Trifluorosilylphosphinidene and Isomeric Phosphasilene and Silicon Trifluorophosphine Complex
Source: Inorg Chem. 2024 Apr 9;63(16):7286–92. doi: 10.1021/acs.inorgchem.4c00135 (PMC11040725; doi:10.1021/acs.inorgchem.4c00135)
Supplement: Supplementary file 1 — ic4c00135_si_001.pdf [file ic4c00135_si_001.pdf]

Supporting Information

Spectroscopic Identification of Trifluorosilylphosphinidene and Isomeric Phosphasilene and Silicon Trifluorophosphine Complex

Guohai Deng†, Marc Reimann‡, Carsten Müller†, Yan Lu†, Martin Kaupp‡, and Sebastian Riedel†*

† Institut für Chemie und Biochemie–Anorganische Chemie, Freie Universität Berlin, Fabeckstrasse 34/36, 14195 Berlin (Germany). E-Mail: s.riedel@fu-berlin.de

‡ Institut für Chemie, Theoretische Chemie/Quantenchemie, Technische Universität Berlin, Sekr. C7, Strasse des 17. Juni 135, 10623 Berlin (Germany).

Table of contents

1. Infrared spectra from co-deposition of silicon atoms with PF_3 in argon S2
2. Difference infrared spectra from co-deposition of Si atoms with PF_3 in argon S3
3. AdNDP chemical bonding and nonbonding pattern of $FPSiF_2$ S4
4. Calculated structural parameters of the higher-energy isomers and the transition states S4
5. Calculated total energies and IR frequencies for various F_3PSi isomers S5
6. The Cartesian coordinates of the studied complexes S6

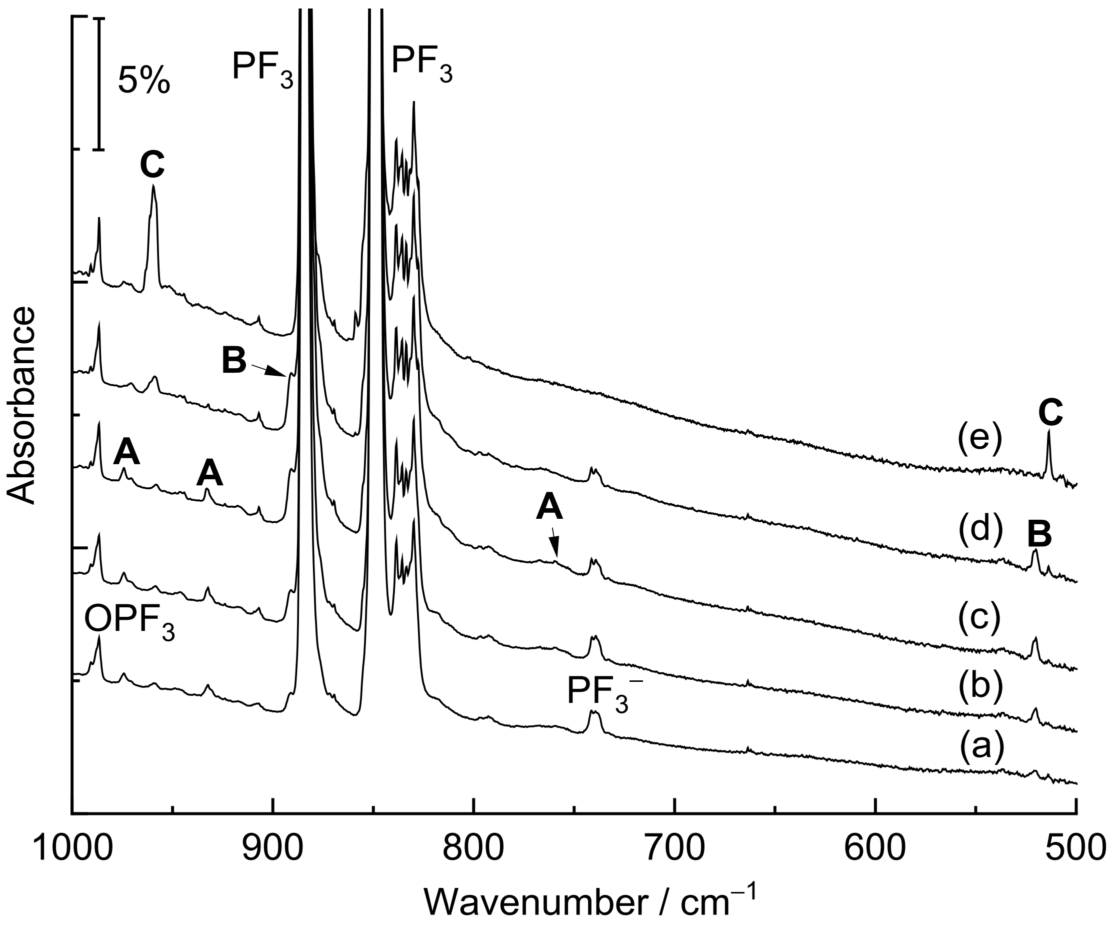

Figure S1. Infrared spectra in the 1000–500 cm^{-1} region from co-deposition of laser-ablated Si atoms with 0.2% PF_3 in argon. (a) After 30 min of sample deposition, (b) after annealing to 15 K, (c) after annealing to 20 K, (d) after 10 min of blue LED (λ = 470 nm) light irradiation, (e) after 10 min of full arc (λ > 220 nm) irradiation. **A**: $FPSiF_2$; **B**: F_3PSi; **C**: F_3SiP.

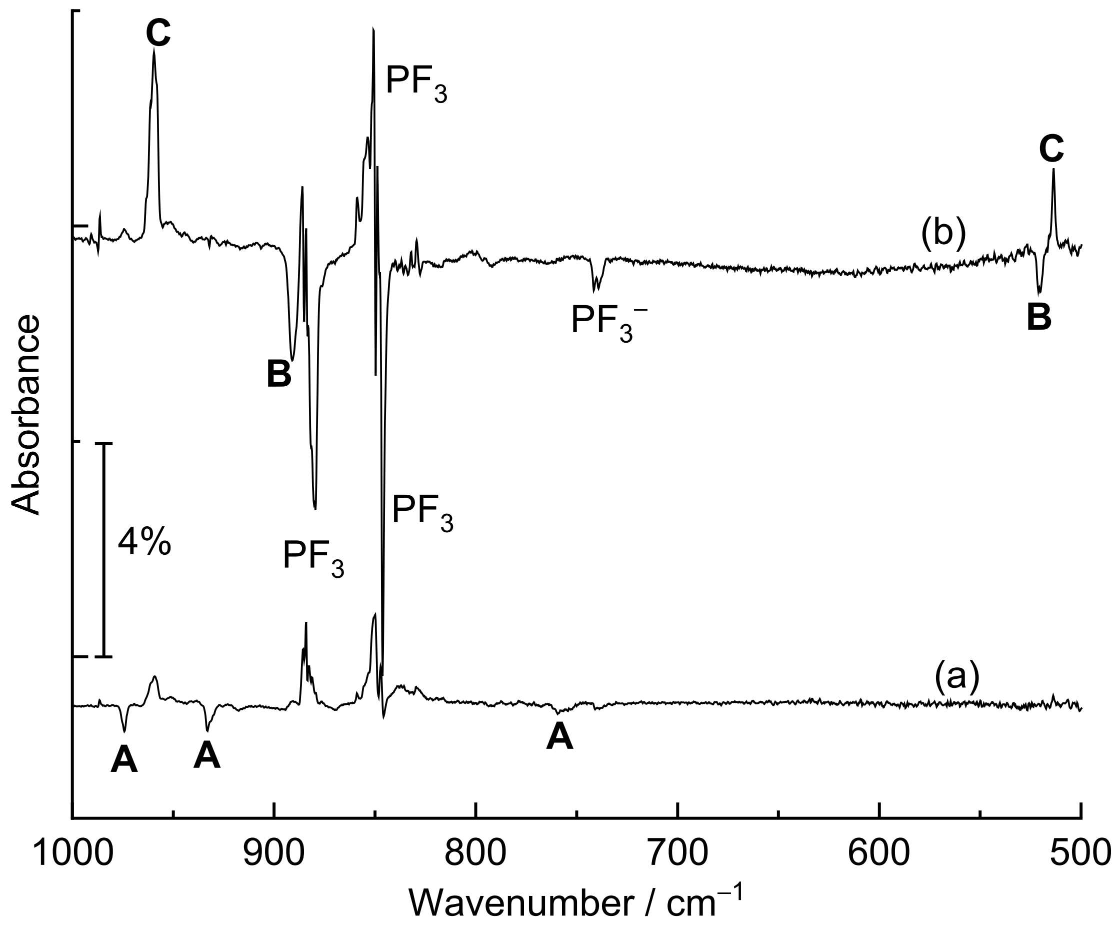

Figure S2. Difference infrared spectra in the 1000–500 cm^{-1} region from co-deposition of Si atoms with 0.2% PF_3 in solid argon. (a) spectrum recorded after 10 min of blue LED (λ = 470 nm) light irradiation minus spectrum recorded after 20 K annealing, (b) spectrum recorded after 10 min of full arc (λ > 220 nm) irradiation minus spectrum recorded after 10 min of blue LED (λ = 470 nm) light irradiation. **A**: $FPSiF_2$; **B**: F_3PSi; **C**: F_3SiP.

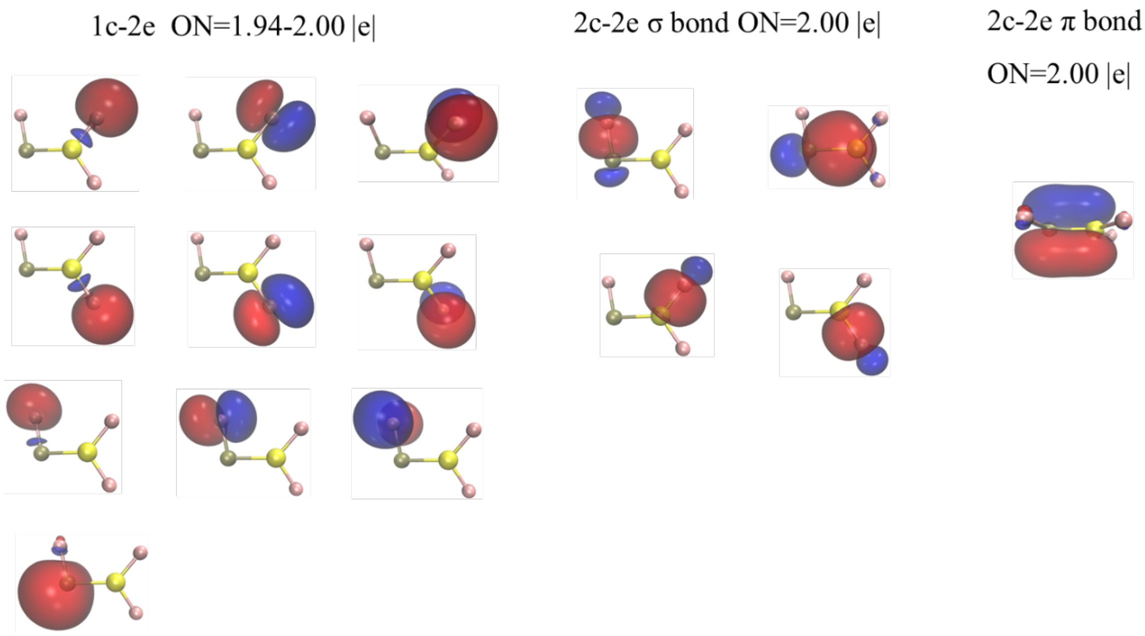

Figure S3. AdNDP chemical bonding and nonbonding pattern of $FPSiF_2$. "ON" stands for occupation number.

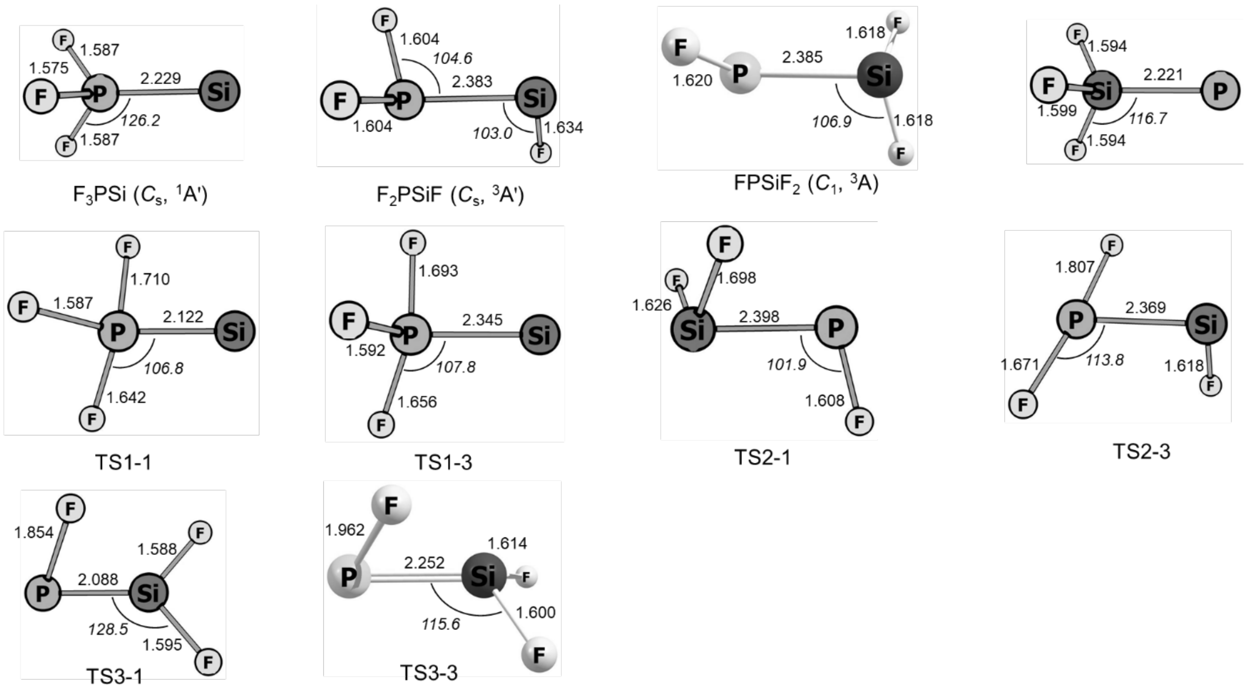

Figure S4. Calculated structural parameters (bond lengths in Ångstroms, bond angles in degrees) of the higher-energy isomers and the transition states at B3LYP/aug-cc-pVTZ level.

Table S1. Calculated total energies, anharmonic IR fundamental frequencies (cm^{-1}) and intensities (km/mol, in parenthesis, left, calculated only for the lower-energy isomers) and harmonic IR frequencies (right) for various F_3PSi isomers at the CCSD(T*)-F12a/ aug-cc-pVTZ-F12 level.

F_3PSi (singlet) *-929.37275224*		F_3PSi (triplet) *-929.39691371*	
	935<br>894<br>857<br>541<br>392<br>342<br>262<br>154<br>95	901 (178.3)<br>888 (134.4)<br>886 (402.7)<br>520 (107.5)<br>358 (4.3)<br>357 (3.8)<br>330 (10.8)<br>172 (1.7)<br>172 (1.7)	914<br>914<br>902<br>527<br>361<br>361<br>337<br>174<br>174
F_2PSiF (singlet) *-929.43555619*		F_2PSiF (triplet) *-929.39555635*	
842 (190.4)<br>803 (124.1)<br>776 (141.7)<br>453 (17.0)<br>349 (8.0)<br>222 (1.1)<br>143 (15.7)<br>93 (1.5)<br>48 (2.1)	852<br>814<br>788<br>460<br>352<br>224<br>143<br>96<br>42		868<br>849<br>847<br>426<br>388<br>258<br>219<br>190<br>130
$FPSiF_2$ (singlet) *-929.47660824*		$FPSiF_2$ (triplet) *-929.44495706*	

977 (195.5)<br>933 (255.0)<br>765 (152.0)<br>521 (0.4)<br>313 (28.0)<br>253 (5.5)<br>195 (12.1)<br>141 (0.8)<br>106 (6.4)	991<br>946<br>775<br>527<br>316<br>255<br>198<br>142<br>106		910<br>864<br>822<br>419<br>347<br>221<br>169<br>150<br>16
F_3SiP (singlet)<br>*-929.49297341*		F_3SiP (triplet)<br>*-929.53210439*	
	982<br>972<br>863<br>523<br>326<br>315<br>306<br>155<br>114	965 (1560.0)<br>964 (660.0)<br>854 (248.9)<br>514 (76.8)<br>317 (48.0)<br>316 (106.0)<br>316 (61.1)<br>175 (4.9)<br>175 (1.7)	978<br>978<br>865<br>518<br>319<br>317<br>317<br>175<br>175

Table S2. The Cartesian coordinates of the studied complexes at the CCSD(T*)-F12a/aug-cc-pVTZ-F12 level.

F_3PSi (C_{3v}, $^3A_1$)			
0 3			
Si	-0.0000000000	0.0000000000	-2.0460624166
P	-0.0000000000	0.0000000000	0.1660956700
F	0.6783709894	1.1749730201	0.9140758798
F	-1.3567419789	0.0000000000	0.9140758798
F	0.6783709894	-1.1749730201	0.9140758798

F_3PSi (C_s, $^{1}A'$)

0 1

Si	0.1646795004	0.0000000000	-2.0261914570
P	0.0459128793	0.0000000000	0.1689332883
F	0.5710126154	1.1395591278	1.0912218126
F	-1.4593846588	0.0000000000	0.5258958892
F	0.5710126154	-1.1395591278	1.0912218126

F_2PSiF (C_1, ^{1}A)

0 1

Si	0.4022348828	-0.6526399161	-1.2136222145
P	-0.5725123901	0.1577224761	0.8072066366
F	-0.3532848322	0.3848782241	-2.1823776776
F	0.6005599306	1.2625054899	0.8927419461
F	0.0937533846	-0.9434788866	1.7608027749

F_2PSiF (C_1, ^{3}A)

0 3

Si	0.5443857640	-0.3425090797	-1.3155032128
P	-0.3792526998	0.3036430801	0.7311326908
F	-0.6038912825	-0.0519184681	-2.4065538673
F	0.7241164906	1.2300884297	1.3743014323
F	-0.1115715858	-0.9290840608	1.6788244443

$FPSiF_2$ (C_s, $^{1}A'$)

0 1

Si	0.0000000000	-0.0076471599	-0.6874438833
P	0.0000000000	-0.7234609203	1.2913858240
F	0.0000000000	0.6986244442	2.0718261380
F	0.0000000000	-0.9728229891	-1.9297939506
F	0.0000000000	1.4649435008	-1.2350981917

$FPSiF_2$ (C_s, $^{3}A''$)

0 3

Si	0.0000000000	0.4104667662	-0.7698664259
P	0.0000000000	-0.6475568236	1.3061753776
F	0.0000000000	0.6736758936	2.2087573842
F	1.2542910009	-0.1111960597	-1.6022801879
F	-1.2542910009	-0.1111960597	-1.6022801879

F_3SiP (C_{3v}, $^3A_1$)			
0 3			
P	-0.0000000000	0.0000000000	-1.9183216547
Si	-0.0000000000	0.0000000000	0.3115729874
F	0.7320765705	1.2679938151	0.8895622593
F	-1.4641531410	0.0000000000	0.8895622593
F	0.7320765705	-1.2679938151	0.8895622593
F_3SiP (C_1, ^1A)			
0 1			
P	-0.0001610716	-0.1086525088	-1.9064460865
Si	-0.0000013099	-0.0064088910	0.3102437749
F	0.0015046030	1.5310290628	0.6569736819
F	-1.2505274930	-0.6709592884	0.9972554622
F	1.2492874196	-0.6734920717	0.9970538672

Table S3. The Cartesian coordinates of the studied complexes at the B3LYP/aug-cc-pVTZ level.

TS1-1			
0 1			
P	-0.25862700	-0.04202100	-0.03476700
Si	1.83787600	-0.28958100	0.17660500
F	-0.88943800	-1.53724700	-0.28771600
F	0.14940200	1.58695800	-0.35708600
F	-1.68783600	0.47078300	0.42802800
TS1-3			
0 3			
P	-0.36392700	0.01839300	-0.26003000
Si	1.89483800	-0.39458900	0.21583700
F	-1.12508000	-1.44419000	-0.41782500
F	0.00615700	1.65029400	-0.51625300
F	-1.22205700	0.37704600	1.03171500
TS2-1			
0 1			
F	-2.01800900	0.74398600	0.51329400
Si	-0.91301500	0.24332700	-0.56887400
P	1.15967000	-0.14555300	0.57231300
F	2.22333100	0.50885200	-0.44071500

F	-0.71785800	-1.38875700	-0.14151800
TS2-3			
0 3			
Si	1.28003700	0.18016300	0.50259800
F	-0.48596300	1.82236900	-0.26653900
F	1.90731600	-0.88132900	-0.54455300
P	-1.02543600	0.11274800	-0.03697900
F	-1.70346200	-1.40920800	0.09090500
TS3-1			
0 1			
F	1.65154900	-1.08773900	0.38752300
Si	0.48154100	-0.13270500	-0.12442000
P	-1.56104700	-0.54155400	-0.26739900
F	-1.07517700	0.98528100	0.66500300
F	1.27631100	1.21147800	-0.41331900
TS3-3			
0 3			
Si	0.52965500	0.03989500	0.15383200
P	-1.58862800	0.58787200	-0.37868700
F	-1.09649400	-0.91851600	0.77809300
F	1.15155200	-1.19028400	-0.65933600
F	1.76874800	1.06695400	0.27309400
